# Nutritional knowledge, attitude and practice of Iranian households and primary health care staff: NUTRIKAP Survey

**DOI:** 10.1186/2251-6581-12-12

**Published:** 2013-02-11

**Authors:** Bahar Azemati, Ramin Heshmat, Maryam Sanaei, Forouzan Salehi, Farzaneh Sadeghi, Maryam Ghaderpanahi, Mojde Mirarefin, Zahra Abdollahi, Mohsen Rezaei Hemami, Bagher Larijani

**Affiliations:** 1 Department of Nutrition, School of Public Health, Loma Linda University, Loma Linda, CA USA; 2 Chronic Diseases Research Center, Tehran University of Medical Sciences, Tehran, Iran; 3 Endocrinology & Metabolism Research Center, Tehran University of Medical Sciences, Tehran, Iran; 4 Ministry of Health, Tehran, Iran; 5 Elderly Health Research Center, Endocrinology & Metabolism Research Institute, Tehran University of Medical Sciences, Tehran, Iran; 6 School of Public Health, Department of Epidemiology & Biostatistics, Tehran University of Medical Sciences, Tehran, Iran

**Keywords:** Nutrition, Knowledge, Attitude, Practice, Household, Health care staff

## Abstract

The aim of this study is to determine knowledge, attitude and practice of Iranian households and health staff on nutrition at province level. The sampling method in NUTRIKAP survey for households in each province is single-stage cluster sampling and the size of clusters is equal. The sampling method for health staff in each province is stratified random sampling. Samples are selected from physicians, health experts, health technicians, nutritionists and health assistants (Behvarz). Overall, 14136 people in 57 clusters in each province and 480 health staff over the country participate in this survey. The necessary data will be gathered by the structured questionnaire and the interview with the eligible person in each household. Data gathering from health staff will be carried out by self-administered questionnaire. The results of this study can help the bureau of community nutrition to provide the proper interventions to improve nutritional health of households.

## Introduction

Eating patterns of Iranian people have changed throughout the past decades. The imbalance dietary pattern can contribute in increasing prevalence of obesity and chronic diseases. Proper interventions can improve eating behavior and build a healthy dietary pattern to prevent diseases. In order to design an appropriate nutrition intervention, it is important to understand the factors associated with individual's food choices, such as nutrition knowledge, attitude and practice [1, 2].

It seems that malnutrition is mostly due to lack of nutritional knowledge rather that food insufficiency. The data of the budget of the family shows that food expenses constitute 41.3% of the whole income of an average urban family, while this ratio is 65% for an average rural family [3]. The results of knowledge, attitude and behavior (KAB) study on nutrition as a part of Nutrition and Health Survey in Taiwan in 2005–2008 indicated that adults’ knowledge on “relationship between diet and disease” and “comparison of foods in terms of specific nutrients” was acceptable. But they lack knowledge on “daily serving requirements” and “weight and weight loss” [4]. The results of the study on knowledge, attitude and practice of Tehranian adults about nutrition revealed that for knowledge 26.5, 52.7 and 20.8%, for attitude, 27.6, 48.9 and 23.5% and for practice, 27.4, 51.7 and 20.9% of individuals had desirable, moderate and weak knowledge scores. This study emphasized that age, gender and education are among the factors that can influence nutritional knowledge, attitude and practice [5].

Primary care physicians can play a role in decreasing morbidity and mortality with proper nutrition counseling. In primary health care (PHC) system, health care staff is the reliable source of nutritional information for patients. Nutrition science changes with new scientific evidences, so it is necessary for health care staff to be aware of reliable sources of continuing nutrition education. A national study in Riyadh among primary care physician indicated that 75% of physicians described their nutritional knowledge as poor [6]. The study on nutritional knowledge of resident physicians in the US indicated that 77% of participants believed that nutrition assessment should be included in routine primary care visits. 14% agreed that physicians were adequately trained to provide nutrition counseling [7].

The bureau of community nutrition has proposed the interventions to improve the nutritional health of the community especially those who are at risk of nutritional problems. This would not happen unless nutritional habits and knowledge, attitude and practice of households, and health care providers on nutrition are determined. The aim of this study is to evaluate knowledge, attitude and practice of Iranian households and health staff on nutrition at province level in 2011 and we hope the results of NUTRIKAP survey can help the bureau of community nutrition to support the proper interventions to improve nutritional health of households.

## Materials and methods

### The target population has two categories

#### Households

The statistical population of the study is ordinary households in rural and urban areas in all provinces of the country. The statistical unit of the study is mother or any member of the household over 15 years of age who is responsible for cooking for the whole family. Non-Iranian households are excluded from the study. Moreover, failing to be present at the time of interview (three times) can also exclude a household from the study.

#### Health staff

The statistical population of the study is health staffs who work in health centers, health units, and health houses in all provinces of the country. The statistical unit of the study is; health staffs who have worked for more than 6 months in PHC network, such as physicians, health experts, nutritionists, family health technicians and health assistants (Behvarz).

## Sampling design

### Households

The sampling method in this survey in each province is single-stage cluster sampling and the size of clusters is equal.

In order to define the optimal size of each cluster (M) and design effect (Deff) for the calculation of the final sample size, based on experts’ opinions Interclass Correlation Coefficient (ρcs) is estimated to be 0.06. In addition, proportion of the expenses to reach to each statistical unit to expenses of data gathering for each member of the cluster $\left(\frac{C_{1}}{C_{2}}\right)$ is estimated to be 4. Therefore, according to this formula $M=\sqrt{\frac{C1}{C2}\times \frac{1-\rho }{\rho }}$, the optimal size of each cluster is estimated to have 8 people, which means there are 8 statistical units in each cluster.

According to this formula *Deff* = 1 + *ρ*_*cs*_(*M* − 1) design effect is estimated to be 1.42, but it is increased to 1.5 in order to achieve more accuracy and to decrease the effect of cluster accumulation. Therefore, the sample size of the study is defined according to the equation N _cs_ = N_srs_ × Deff_cs_. In this equation Nsrs is the calculated sample size for simple random sampling and Ncs is the real sample size for cluster sampling with a design effect equal to Deffcs.

### Health staff

The sampling method in this survey in each province is stratified random sampling. Samples are selected from physicians, health experts, health technicians, nutritionists and health assistants (Behvarz). Number of samples in each stratum is defined according to the proportion to size in each stratum, using the formula N_i_ = N_srs_ × D_i_. Di is density or proportion in each stratum and Ni is the number of samples in the same stratum. Thereafter, samples are selected in each stratum by systematic random sampling. The sampling interval is equal $I=\frac{\mathrm{Si}}{\mathrm{Ni}}$. Si is the total number of people in each stratum and Ni is the number of samples of the study in the same stratum. The first sample is determined using random numbers between 1 and I.

## Sample size

### Households

The sample size for the study is 385 households based on the equation $N_{\mathrm{srs}}=\frac{Z^{2}1-\alpha /2\;\left[P\times \left(1-P\right)\right]}{d^{2}}$ for simple random sampling. Therefore, regarding design effect of 1.5, the sample size for cluster sampling is calculated to be 385 (57 clusters). Overall, 14136 people (in 31 provinces) participated in the study. Based on previous studies, the probability of type I error (α) is 5% and the proportion of proper knowledge over the target population and estimated accuracy is 40% and 6%, respectively. (Relative accuracy of 15%).

### Health staff

The sample size for health staff equals to 432 people in all provinces. This sample size is calculated for the estimated proportion of proper knowledge to be 80% and estimated accuracy to be 12% and considering the probability of type I error to be 5%. Regarding the response rate to be 80%, the sample size is increased to 480 people.

### Tools for data gathering

There are 27 questions in knowledge, 32 questions in attitude and 30 questions in practice section (including food frequency questions) of the questionnaire. The answers of knowledge questions are scored dichotomously. In attitude section of the questionnaire, a range of response options are used including five point Likert scales. Theme of the questionnaire is around the basic principles of nutrition, food groups, sources of nutrients, diet-disease relationship, and nutritional requirement at different life stages.

### Households

The necessary data will be gathered by the structured questionnaire and the interview with the eligible person in each household.

### Health staff

Data gathering from physicians and nutritionists will be completed through self-administered questionnaire. Data gathering from health technicians and assistants (Behvarz) will be carried out based on the structured questionnaire and interview.

Evaluation of the validity and reliability of the questionnaire: the pilot study

A number of demographic questions are included in the survey to characterize respondents. A literature review of existing questions is carried out and 13 items chosen. These ask about sex, age, marital status, number of children, educational level, occupation and partner's occupation, the ownership of the house and etc. Some items of the nutrition questionnaire are taken from existing questionnaires while others are generated with expert advice from a panel of nutritionists, epidemiologists and health educators. This process serves to maximize the content validity of the questionnaire [8, 9]. Using the pool of 116 items, two reviews are carried out by the panel to select the best in terms of clarity, accuracy and interpretability of the questions. The questionnaire is piloted in a convenience sample of the households (one cluster) and the health staff (nutritionists). After completing the questionnaire, they are asked about the content of questionnaires. Review of the completed surveys and subjects’ comments by another team of experts lead to minor changes to the tool to improve comprehension and ability to respond.

Cronbach's alpha is used to assess the reliability of the scores with respect to (a) how well the individual items of the question fit together and (b) whether they assess the same construct. It is 0.79 and 0.80 for households and health staff, respectively.

The ability of each individual item to discriminate between people with different levels of knowledge is measured by correlating the score on each item with the overall test score. As mentioned before, to test construct validity of the final version of the questionnaire, we administer it to one cluster of households and nutritionists as health staff to determine if the questionnaire can differentiate different levels of nutritional knowledge. A significant difference in knowledge is found between two groups (Mann–Whitney statistic Z= − 8.05, P < 0.01). To validate practice, we use a three-day food record using Spearman correlation (r = 0.83, P< 0.001).

One of the main strengths of this study is the large sample size. Our subjects are representative of general population in urban and rural areas in each province.

### Quality assurance

Quality assurance has two parts; 1) in designing phase; tools for data gathering are standardized and are pilot tested for validity and reliability. The operations manual of the study is evaluated and revised in the pilot study. All interviewers and supervisors are briefed about the different phases of the study. 2) The protocol is given to all users and supervisors. Each phase of the study will be monitored by supervisors at the province and national levels. At the end of the sampling, the monitoring officers in each province will control the questionnaires and in case of incomplete questionnaire, they will return them to the interviewers for further clarification and modification of any missing or inconsistent information. The auditors also will control the sampling process and randomly will recheck 10% of the questionnaires of each province to identify any errors.
